# A novel assessment of the traction forces upon settlement of two typical marine fouling invertebrates using PDMS micropost arrays

**DOI:** 10.1242/bio.030262

**Published:** 2017-12-14

**Authors:** Kang Xiao, Wen-Bin Cao, Cu-Huang Rong, Lian-Guo Chen, Xiao-Xue Yang, Wei-Jia Wen, Pei-Yuan Qian, Zhang-Li Hu, Ying Xu, Yu Zhang

**Affiliations:** 1 Shenzhen Key Laboratory of Marine Bioresource and Eco-environmental Science, Guangdong Engineering Research Center for Marine Algal Biotechnology, College of Life Sciences and Oceanography, Shenzhen University, Shenzhen 518060, P.R. China; 2 Key Laboratory of Optoelectronic Devices and Systems of Ministry of Education and Guangdong Province, College of Optoelectronic Engineering, Shenzhen University, Shenzhen 518060, P.R. China; 3 Department of Physics, The Hong Kong University of Science and Technology, Clear Water Bay, Kowloon, Hong Kong SAR, P.R. China; 4 Division of Life Science, The Hong Kong University of Science and Technology, Clear Water Bay, Kowloon, Hong Kong SAR, P.R. China

**Keywords:** Marine biofouling, Barnacle, Bryozoan, Metamorphosis, PDMS micropost arrays, Traction force

## Abstract

Marine biofouling poses a severe threat to maritime and aquaculture industries. To prevent the attachment of marine biofouling organisms on man-made structures, countless cost and effort was spent annually. In particular, most attention has been paid on the development of efficient and environmentally friendly fouling-resistant coatings, as well as larval settlement mechanism of several major biofouling invertebrates. In this study, polydimethylsiloxane (PDMS) micropost arrays were utilized as the settlement substrata and opposite tractions were identified during early settlement of the barnacle *Amphibalanus amphitrite* and the bryozoan *Bugula neritina*. The settling *A. amphitrite* pushed the periphery microposts with an average traction force of 376.2 nN, while settling *B. neritina* pulled the periphery microposts with an average traction force of 205.9 nN. These micropost displacements are consistent with the body expansion of *A. amphitrite* during early post-settlement metamorphosis stage and elevation of wall epithelium of *B. neritina* during early pre-ancestrula stage, respectively. As such, the usage of micropost array may supplement the traditional histological approach to indicate the early settlement stages or even the initiation of larval settlement of marine fouling organisms, and could finally aid in the development of automatic monitoring platform for the real-time analysis on this complex biological process.

## Abstract (precis)

**Summary:** The traction forces of two typical marine fouling invertebrates were quantitatively assessed by using PDMS micropost arrays and beam-bending theory.

## INTRODUCTION

Marine biofouling refers to the undesirable accumulation of living organisms including microorganisms, seaweeds, and animals on submerged surfaces (Callow and Callow, 2002). Among which, fouling invertebrates cause major damage to ship hulls and propulsors, block mariculture cages and water pipes, increase fuel consumption of marine transport, and therefore are one of the most serious problems of maritime and aquaculture industries (Yebra et al., 2004; Schultz et al., 2011; Clare, 1995). For instance, US navy vessels spend 180 to 260 million US dollars annually to control and prevent biofouling (Schultz et al., 2011). Historically, a variety of physical and chemical antifouling methods have been implemented (Qian et al., 2013). In particular, the most commonly employed practice is to coat submerged surfaces with biocidal paints, such as organotin, tributyltin (TBT), Irgarol 1051 and Sea-Nine 211 (Qian et al., 2013). Due to the threat to the marine environment revealed after a long-term application, e.g. toxicity on non-target marine organisms, some biocides were banned or restricted in recent years (Cresswell et al., 2006; Qian et al., 2013). The development of effective and environmentally friendly antifouling technique requires a more in-depth understanding of environmental risk and working mechanisms of antifouling compounds.

In previous studies, multiple omics and biological approaches have been utilized to reveal the detailed developmental process (Qian et al., 2013), the key biomarkers and major signaling pathways in larval settlement of the major marine biofouling species, including the barnacle *Amphibalanus amphitrite*, the polychaete *Hydroides elegans* and the bryozoan *Bugula neritina*. In particular, the temporal and spatial metamorphic changes of fouling organisms were mainly examined by histological studies. Through these, the unique developmental patterns of different species, such as *A. amphitrite* (Bernard and Lane, 1962; Maruzzo et al., 2012) and *B. neritina* (Reed and Woollacott, 1983; Wong et al., 2012), have been revealed. Furthermore, the histological staining, which showed the anatomy of *B. neritina* during metamorphosis, served as the reference map to precisely interpret the spatial gene expression patterns and the involvement of certain signaling pathways underlying metamorphosis (Wong et al., 2012). Although the current techniques such as light sheet microscopy have enabled the non-destructive slide-free observations on thick intact tissues or organisms (Cella Zanacchi et al., 2011; Cresswell et al., 2006), the histological studies require a complex sample preparation and data acquisition process and cannot be analyzed in a real-time manner. Therefore, supplementary simple and real-time approaches are demanded for high-throughput developmental pattern exploration of marine fouling organisms.

Researchers have adopted a variety of physical and chemical approaches to examine the settlement preference, strength and elemental characterization for a better understanding of the settlement mechanisms. For instance, the adhesion strength of adult *A. amphitrite* has already been measured by applying a mechanical shear force to the organism base (Swain et al., 1992; Conlan et al., 2008). To overcome the drawbacks of the shear-force method, Aldred et al. assessed the attachment strength of *A. amphitrite* cyprids by a calibrated water jet, and the settlement preference of *A. amphitrite* on the textures sized from 0 to 512 μm on PDMS microtextured matrix was also examined (Aldred et al., 2010). Also, protein deposits of *A. amphitrite* during surface exploration on model surfaces with variable hydrophilicities were studied by Atomic Force Microscope, revealing a positive relationship between protein deposits and hydrophobicity (Guo et al., 2014). More recently, the elemental distributions during the early attachment phase of *A. amphitrite* larvae and juvenile *A. amphitrite* were established by synchrotron radiation-based μ-XRF, which may inspire further functional investigations on the previously unrecognized elements, including bromine, iron, and selenium identified in the cyprid permanent adhesive (Senkbeil et al., 2016). However, the assessment on the dynamic morphological changes of settling fouling organisms using physical approaches has not been reported.

Because of the advantages in mechanical application and quantitative measurements, PDMS micropost arrays have been employed in a wide spectrum of biological and chemical studies, including assessing macromolecular transport (Chien et al., 2017) and designing dielectrophoretic trapping (Mohammadi et al., 2016). Comparable studies were performed to assess the mechanics of adhesive mammalian cells and to clarify the correlation of biochemical and mechanical signals (Beussman et al., 2016; Rodriguez et al., 2014; Tan et al., 2003; Saez et al., 2007). In particular, the contractile forces of cardiomyocytes were measured through calculating the twitch force of bending cylindrical posts by beam-bending theory (Beussman et al., 2016; Rodriguez et al., 2014). Therefore, in this study, PDMS micropost arrays were utilized as the settlement substrata to assess the horizontal movements caused by morphological changes of settling fouling organisms. The assessment results may assist to develop a real-time observation method supplementing traditional histological approaches.

## RESULTS

### Different basal tractions upon *Amphibalanus amphitrite* and *Bugula neritina* settlement

**Fig. 1.**
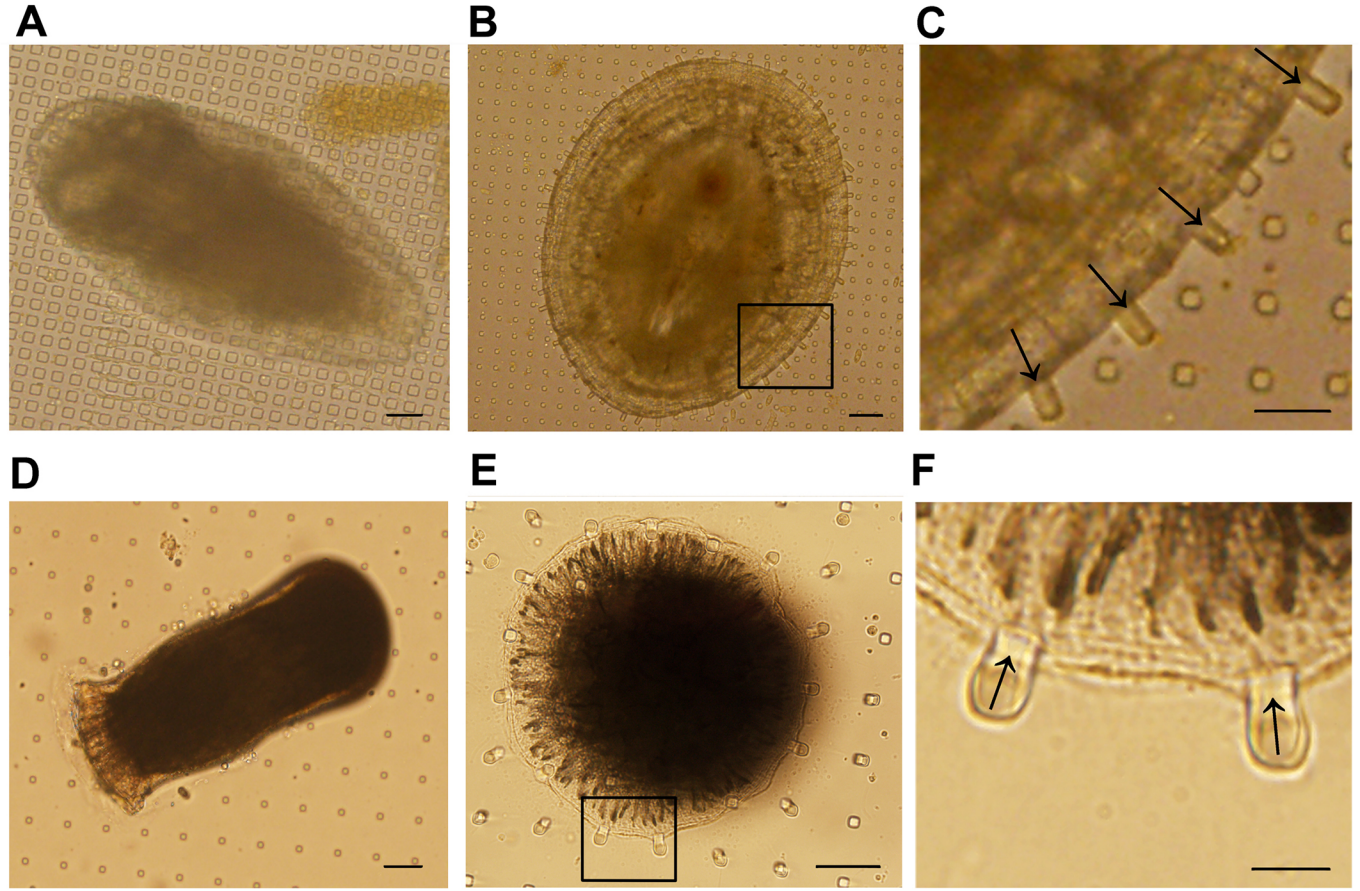
**Microscopic images of settling the barnacle *A. amphitrite* and the bryozoan *B. neritina* cyprids.** (A) Early settled *A. amphitrite* cyprid. (B) Settled *A. amphitrite* juvenile. (D) Early pre-ancestrula 4 h stage of *B. neritina*. (E) Early pre-ancestrula 1 h stage of *B. neritina*. The regions marked with rectangles in B and E were enlarged and shown in panels C and F, respectively. The arrows indicate the directions of micropost tip displacements. Micropost intervals: 40 μm (A), 35 μm (B,C), 15 μm (D) and 20 μm (E,F). Scale bars: 40 μm (A), 35 μm (B), 10 μm (C), 15 μm (D), 40 μm (E) and 20 μm (F).

**Fig. 2.**
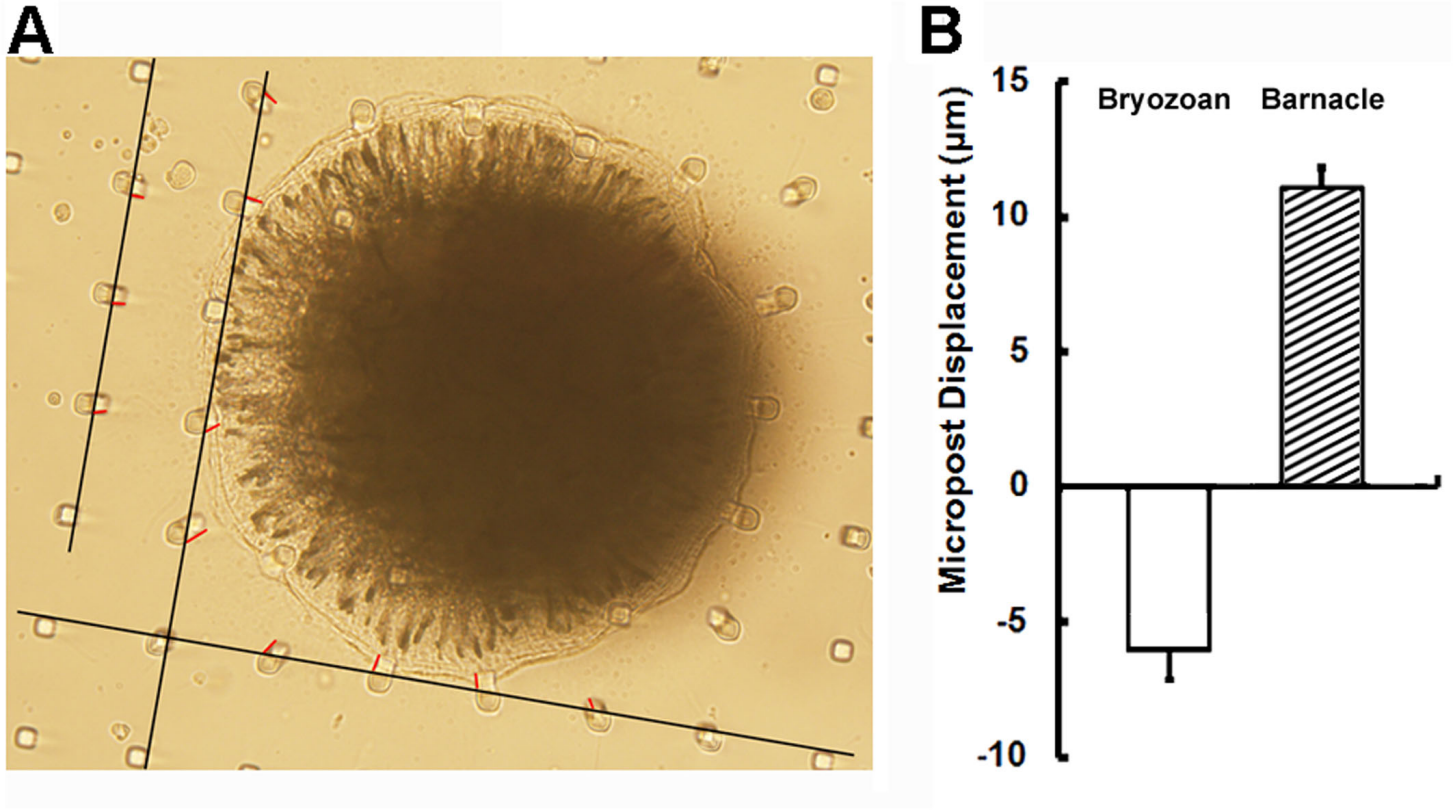
**The quantitative analysis on PDMS micropost displacements caused by settling the barnacle *A. amphitrite* and the bryozoan *B. neritina* cyprids.** (A) A figure illustrating how micropost displacements were measured. Black and red lines represent micropost alignment for determination of initial positions and micropost displacements respectively. Post intervals: 35 μm. (B) The comparison of micropost displacements caused by settling *A. amphitrite* and *B. neritina* cyprids. Data is displayed as mean±s.d.

In both *A. amphitrite* and *B. neritina*, displacements of micropost tips surrounding the settling individuals were observed (Fig. 1). However, the directions of displacements in these two fouling species were opposite. The settled *A. amphitrite* pushed the periphery microposts in the centrifugal direction (Fig. 1B,C). In contrast, the settled *B. neritina* pulled the periphery microposts in centripetal direction (Fig. 1E,F). To ensure the accuracy of the measurement of tip displacement, the initial tip positions of displaced microposts were determined by the alignment (dark lines) of micropost tips, and then the displacements were indicated in red lines by the comparison between initial and displaced positions and measured twice by ImageJ (Fig. 2A). As measured from hundreds of tilt microposts, the absolute values of micropost tip displacements caused by settled *A. amphitrite* and *B. neritina* were 11.00±0.84 μm (*n*=188) and 6.02±1.19 μm (*n*=277), respectively (Fig. 2B). Assuming that the outward micropost displacement and the pushing force of settling *A. amphitrite* individuals are positive values, then the inward micropost displacement and the pulling force of settling bryozoan individuals are negative. As calculated according to formula (Eqn 8), the average traction forces of settled *A. amphitrite* and *B. neritina* were 376.2 nN and −205.9 nN, suggesting *A. amphitrite* may exert much stronger traction force than bryozoan. The values of traction forces of settled fouling species are 10 times more than the reported contractile forces of human induced pluripotent stem cell-derived cardiomyocytes (hiPSC-CMs) (Rodriguez et al., 2014), which is possibly due to the difference in the body mass of different organisms.

**Fig. 3.**
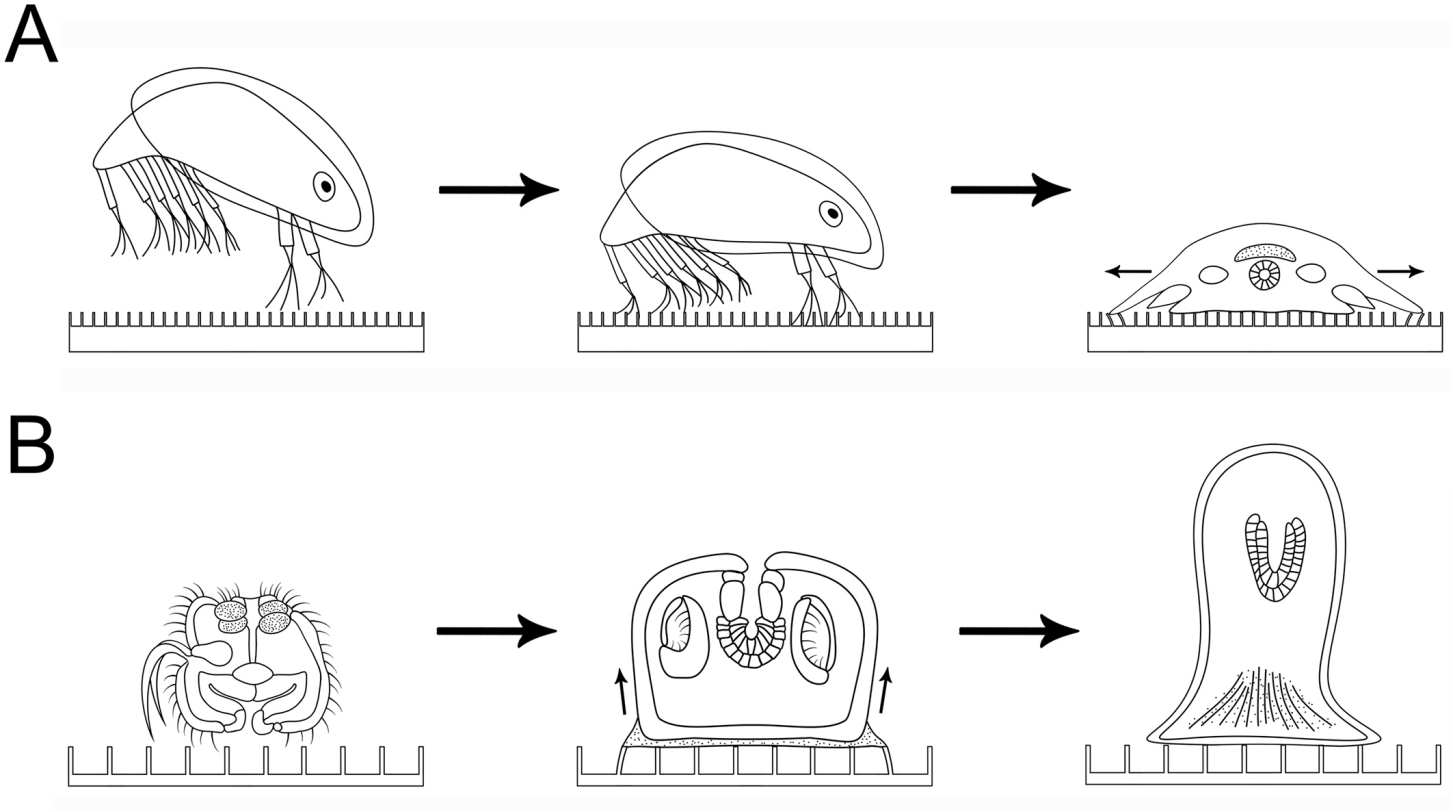
**Diagrams of the correlation between micropost displacements and the metamorphic changes of *A. amphitrite* and *B. neritina* cyprids during early post-attachment development.** (A) Diagrams showing *A. amphitrite* cyprids developed from swimming stage (left panel, side view), early settlement stage (middle panel, side view) to juvenile (right panel, front view). Micropost interval: 20 μm. (B) Side view diagrams showing *B. neritina* cyprids developed from swimming stage (left panel) to two pre-ancestrula stages (middle and right panels). The arrows indicate the elevation of wall epithelium. Micropost interval: 35 μm.

Images of settling *A. amphitrite* individuals were captured by stereomicroscope at 48 h after sample loading (juvenile stage). As shown in Fig. 1A,B, two types of morphologies were observed among the settling *A. amphitrite* individuals, which could be classified into early settlement stage (also called ‘settled pad’, Fig. 1A) and juvenile stage (Fig. 1B), and this is due to the asynchronous development of each individual. In contrast to bryozoan, only *A. amphitrite* individuals in juvenile stage but not in early settlement stage pushed the periphery microposts away from their own bodies, as micropost displacement could only be observed in the juvenile stage but not in the early settlement stage. In early settlement stage, *A. amphitrite* cyprids loosely attached to the substrata and body expansion was not observed (Fig. 3A, middle panel). At 48 h post sample loading, the just-settled individuals developed into juveniles with obvious body expansions in bilateral directions. The periphery microposts were centrifugally pushed by the expanding body, leading to the observation of outward micropost displacements (Fig. 3A, right panel). As such, metamorphic difference between two developmental stages can be identified and distinguished by micropost displacement.

Similarly, settling *B. neritina* individuals were imaged by stereomicroscope at 4 h after sample loading, which were within the early pre-ancestrula stage according to the previous reported histological study (Wong et al., 2012). As shown in Fig. 1D,E, two types of morphologies were observed from the settling individuals, which could be classified into early pre-ancestrula 1-h stage (Fig. 1E) and early pre-ancestrula 4-h stage (Fig. 1D). Interestingly, only individuals in the early pre-ancestrula 1-h stage, but not 4-h stage, pulled the periphery microposts toward their own bodies. At early pre-ancestrula 1-h stage, the wall epithelium rose slowly over the aboral hemisphere (Reed and Woollacott, 1983), so the periphery microposts were centripetally pulled by the rising tissue, leading to the observation of inward micropost displacements (Fig. 3B, middle panel). In early pre-ancestrula 4-h stage, the elevation of wall epithelium has completed, therefore the periphery microposts were no longer pulled and no displacement could be observed (Fig. 3B, right panel). Therefore, these observations agree well with those of the previously reported histology studies and reveal the relation of micropost displacement and the metamorphic change of settling *B. neritina* individuals.

## DISCUSSION

### Rectangular posts versus cylindrical posts

Polydimethylsiloxane micropost arrays have already been utilized to assess the contractile forces of cardiomyocytes (Beussman et al., 2016; Rodriguez et al., 2014). However, these studies employed cylindrical microposts instead of rectangular microposts used in the current study. As the influence of twitch force angle needs to be taken into consideration, the calculation of the twitch forces in this study becomes more complicated. Project the twitch force F_xy_ to the *x*-axis and the *y*-axis to create F_x_ and F_y_ in perpendicular directions. Regardless of the micropost shape, the relation formula between twitch force F_xy_ and F_x_ is
(1)
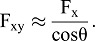
The theoretical maximum influence of twitch force angle lies in F_xy_, in which twitch force angle *θ* is equal to 45°. Therefore, the maximum F_xy_ approximates to 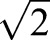
F_x_ and the maximum error to calculate F_xy_ by Eqn 1, in which the twitch force angle was ignored is roughly 41%.

On the other hand, as shown in Fig. 5B, the range of F_xy_ can be determined as F_x_=F_y_<F_xy_<F_D_, in which F_D_ is the twitch force of a cylindrical micropost with the diameter D of the cross section equal to 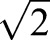
L. F_x_ and F_y_ are calculated by Eqn 7. Therefore, the relation formula between F_D_ and F_x_ is
(2)
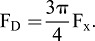
Thus F_D_ is roughly 2.36 times over F_x_, while F_xy_ is less than 2.36 times of F_x_. Since the standard deviations are less than 20% of the measured displacement values, the influence of twitch force angle did not significantly reduce the importance of the estimation/calculation method of this study and the drawback of rectangular posts compared with cylindrical posts can be neglected. Yet, cylindrical posts will be used in future experiments to improve the accuracy of the estimation and calculation.

### Large deflections versus small deflections

The tip deflection ratio βx is introduced as follows:
(3)
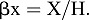
The measured micropost tip displacements caused by settling *B. neritina* and *A. amphitrite* were roughly 6 and 11 μm, so the deflection ratios were 24% and 44% respectively. Eqns 4 to 8, based on beam-bending theory, can merely be applied for small deflections within the linear range. Therefore, a proper non-linear modification for calculating large deflections should be employed. With regard to the evaluation of large deflections of cantilever beams, a few studies have proposed analytical analysis of the nonlinear deformation of beams based on elliptic integrals and functions in a variety of complex ways (Kimiaeifar et al., 2014; Ghaffarzadeh and Nikkar, 2013). However, according to Beléndez's work, when βx are 24% and 44%, the corresponding deviations between the linear approximations and non-linear exact solutions are roughly 7.6% and 24.1% (Beléndez et al., 2002). Therefore, the importance and accuracy of the calculation method of this study were not significantly reduced although the micropost deflections caused by settling *B. neritina* and *A. amphitrite* were beyond linear range.

Alternatively, small deflections caused by settling fouling organisms can be realized by applying PDMS microposts with increased size or stiffness in the future work. Thus, the linear calculation method will be applicable. Furthermore, the most accurate approach is to measure the relation curve between exerted forces and micropost displacements, and then simulate the calculation equation, so the certain exerted forces corresponding to micropost displacements can be calculated accordingly. Through this, the accuracy of the calculated traction forces will be remarkably enhanced.

### Advantages of PDMS micropost in assessing the dynamic settlement

Polydimethylsiloxane has also been successfully utilized in the study of marine fouling organisms, e.g. the settlement preference of marine fouling organisms on PDMS with different texture profiles and sizes was examined (Aldred et al., 2010; Vucko et al., 2014). However, most of these studies did not focus on the dynamic post-attachment metamorphic changes of marine fouling organisms. In this study, we proposed a novel application of PDMS chips or devices in the fouling mechanism studies. Compared with the abundance of technical approaches in cell experiments, tools for studying marine biofouling organisms are very limited, mainly due to the poor understanding of genetic information and signaling pathways, as well as the auto-fluorescence in many marine invertebrates. For instance, fluorescent microposts and cellular biomarkers were both used to enhance the convenience and accuracy of the assessment on cardiomyocytes (Beussman et al., 2016; Rodriguez et al., 2014). However, this may be unfeasible for assessing *B. neritina* or *A. amphitrite* because the observed strong auto-fluorescence of *B. neritina* or *A. amphitrite* (Kamiya et al., 2012) will conceal the fluorescent microposts underneath settling individuals. Nevertheless, PDMS micropost array used in the current study possesses many advantages as compared with other platforms. For example, the observation of micropost displacement and the following calculation of traction force have allowed us to determine the settlement status of two representative fouling species, which have distinct morphological and histological changes. In addition, the transparent nature of PDMS makes it possible to record the morphological changes from both the dorsal and ventral sides. As such, real time imaging the morphological changes from both dorsal and ventral views of the settling organism could be implemented in future studies, to improve the quantitative accuracy and broaden the applications of this PDMS chip.

### The prospective applications of PDMS micropost array in marine biofouling and antifouling studies

This study represents the first report of calculating the traction force of the common settling marine biofouling invertebrates (the bryozoan *B. neritina* and the barnacle *A. amphitrite*) during early post-attachment metamorphosis. The findings in this study may assist the design of a powerful real-time platform for both fouling mechanism studies and anti-fouling drug screening. In particular, in the mechanism studies, micropost displacements caused by the settling of individuals at certain time points can serve as temporal markers to indicate the corresponding developmental stages of both explored and unexplored fouling species. On basis of this, the application of micropost, together with imaging and recording techniques, can serve as an automatic platform to calculate the settlement rate of fouling invertebrates, and subsequently being used as a high-throughput system to determine the efficiency and/or toxicity of antifouling chemicals.

### Conclusions

In summary, the settling bryozoan *B. neritina* and the barnacle *A. amphitrite* in early settlement stages caused opposite displacements of PDMS microposts, which were correlated with the respective wall epithelium elevation and body expansion during their early post-attachment metamorphosis. This method may provide an intuitional tool to supplement the traditional histological approach for a mechanism study of marine biofouling organisms, and to assist the development of a powerful real-time analysis platform for anti-fouling drug screening in the future.

## MATERIAL AND METHODS

### Design and fabrication of PDMS micropost arrays

**Fig. 4.**
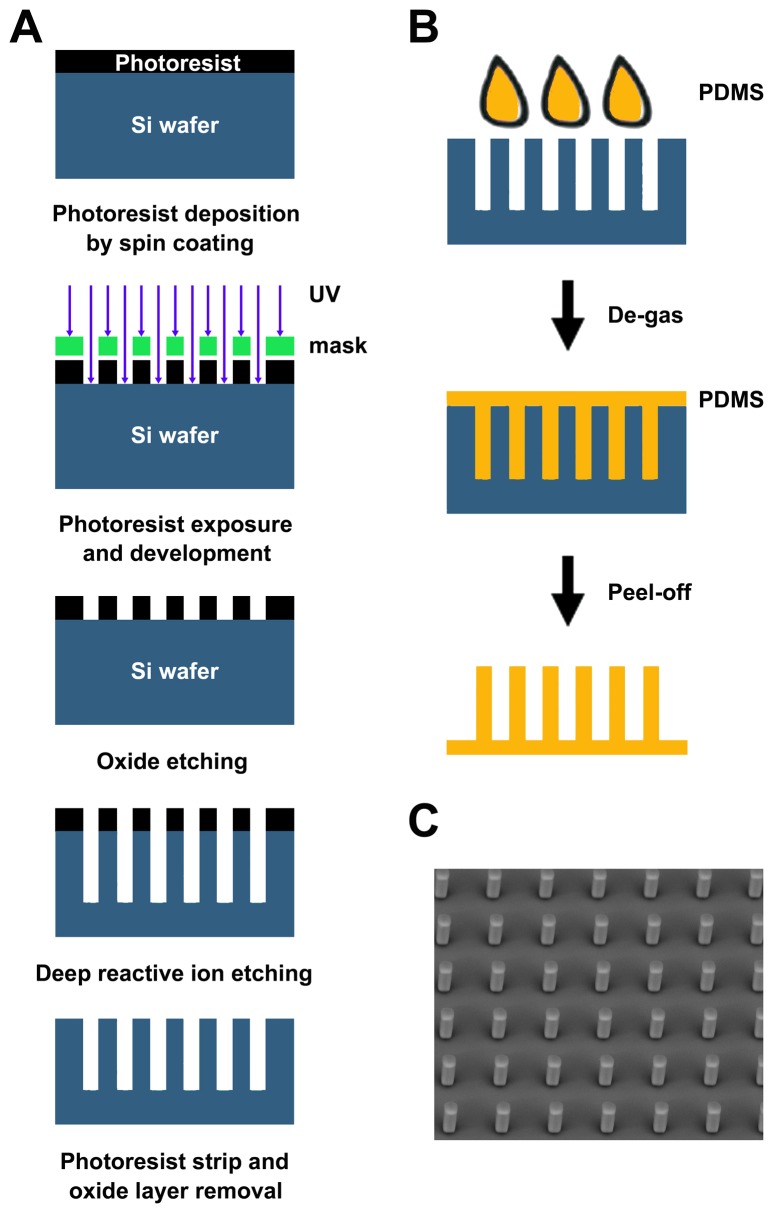
**Flowchart of PDMS micropost array fabrication.** (A) Silicon mold fabrication process. (B) Fabrication of PDMS microposts on silicon mold. (C) A representative SEM image of fabricated PDMS micropost array. Post interval: 25 μm.

The 25-μm height micropost substrate was fabricated on a silicon wafer by the standard photolithographic methods (Duffy et al., 1998; Zhou et al., 2015). The fabrication processing flow charts of the patterned Si mold and PDMS micropost arrays were illustrated in Fig. 4. Briefly, the mold with an integrated array of 5-microwell was fabricated by standard UV photolithography and deep reactive-ion etching (DRIE) on a clean silicon wafer. The depth of the microwell was etched to ∼25 μm. Next, the silicon mold was silanized with (tridecafluoro-1,1,2,2-tetrahydrooctyl)-1-trichloro silane for days in a sealed vacuum chamber. After UV exposure (SUSS Microtec MA6, Garching bei München, Germany) of photoresist on Si wafer and pattern developing, the oxide layer without photoresist cover was further etched by the advanced oxide etch (STS-AOE Etcher, Surface Technology Systems, Coventry, UK), and undergone deep reactive ion etching (ICP DRIE, Surface Technology Systems, Coventry, UK) to generate vertical wells. Finally, the photoresist layer and oxide layer were removed by plasma cleaning process (PS210 Photo Resist Asher) and oxide etcher respectively. Then, as shown in Fig. 4B, PDMS was produced with Sylgard 184 silicone elastomer mixture (Dow Corning Corporation, Miland, USA) at a Base to Curing agent ratio of 10:1. This PDMS mixture was then slowly poured over the patterned region of the mold and placed in a vacuum oven for degasification. The PDMS and the mold were then placed on the hotplate to cure at 70°C for 4 h. After curing, the PDMS layer was gently peeled off in ethanol solution and kept inside the ethanol for ease of releasing and sustaining the microposts at the same time. The PDMS chip was then dried in a CO_2_ supercritical point dryer machine (Automegamdri-916B series C, Tousimis) to prevent the microposts from collapsing during the drying process induced by the capillary force. Finally, each PDMS micropost has a cross section length of either 5 or 10 μm and a height of 25 μm. And the center-to-center interval of 20, 25, 35 or 45 μm was fabricated for assessment. A representative SEM (scanning electron microscope, Tabletop Microscope TM3000, Hitachi, Tokyo, Japan) image of PDMS micropost array is shown in Fig. 4C. The Young's modulus of as-fabricated PDMS was measured by MTS electromechanical universal testing machine CMT4204 (Tianjin, China).

### *Amphibalanus amphitrite* and *Bugula neritina* larval sample preparation

*A. amphitrite* larval sample was prepared following procedures of Zhang et al. (2010). Briefly, adult *A. amphitrite* were collected from the concrete pier posts at Pak Sha Wan in Hong Kong (22°21′45″N, 114°15′35″E), the released naupliar larvae induced by light were kept in 0.22-μm filtered seawater (FSW) for 3 h. The nauplii developed into cyprids on day 4 when reared on a diet of *Chaetoceros gracilis* Schuttat (1.0×10^6^ cells ml^−1^) at 28°C. Once the larvae reached the cyprid stage, they were transferred into a petri-dish containing PDMS micropost arrays immersed in FSW (roughly 50 cyprids per square centimeter).

*B. neritina* larval sample was prepared according to the procedures of Wong et al. (2012). Briefly, adult colonies of *B. neritina* were collected from the floating rafts of a fish farm in San Shing Wan, Hong Kong (22°21′19″N, 114°16′15″E) and maintained in an aquarium with 21°C flow-through seawater for less than 7 days before use. Adults were induced to spawn under a bright artificial light and the larvae were collected for experiments using a mesh with 100-μm pore size (Wang et al., 2010; Wong et al., 2012). The sample loading process was similar to that of *A. amphitrite*.

### Image acquisition and analysis

Three experimental replicates were performed. The images of *B. neritina* and *A. amphitrite* settlement experiments were taken by a stereomicroscope (Olympus IX61, Tokyo, Japan) at 4 h and 48 h after sample loading, respectively. The displacements of micropost tips were measured by comparing their current and initial positions. The acquired images were further processed by Adobe Photoshop (Adobe Systems), and the value of microposts displacement was measured and analyzed by ImageJ (National Institutes of Health, NIH). Quantitative data in the text were reported as mean±standard error.

### Calculation of the traction forces by beam-bending theory

**Fig. 5.**
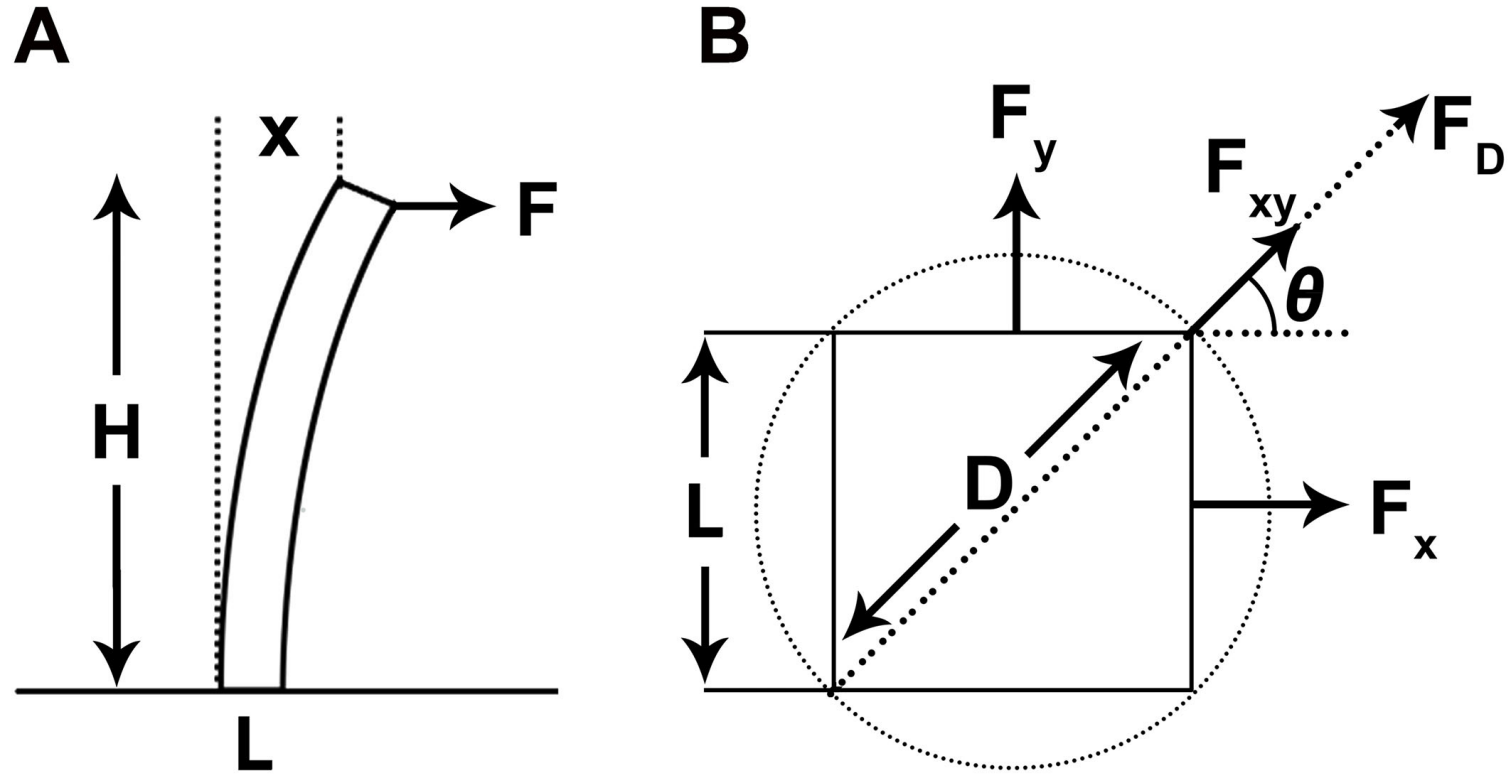
**Diagrams of calculation of micropost twitch force by beam-bending theory.** (A) Diagram of a tilt micropost caused by the point load at the free end exerted by settling cyprid. L is the cross section length of microposts (5 μm), H is the height of microposts (25 μm), X is the tip displacement of microposts, and F is the twitch force. (B) Diagram of the comparison of twitch forces in different directions or micropost shapes. F_x_ and F_y_, equal to F in (A), represent the twitch forces in perpendicular directions with the force angle of 0° or 90°. F_xy_ represents the twitch forces with a force angle *θ* (0°<*θ*<90°). F_D_ is the twitch force of a cylindrical micropost with the diameter D of the circular cross section which is equal to 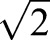
L.

At 4 h and 48 h after sample loading, *B. neritina* and *A. amphitrite* settled on top of the micropost arrays and underwent dramatic morphological changes, and consequently drove the tilt of the microposts. As the settling organisms merely contact the top of the microposts, microposts are modeled as cantilever beams which are fixed at one end and undergo deflections at the free end, the pulling or pushing force (or the point load) at the free end can be calculated by beam-bending theory. The reported calculation of twitch force of bending cylindrical post was performed by following equation (Rodriguez et al., 2014):
(4)
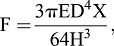
where D is the diameter, E is the Young's modulus (3.42 MPa), H is the height (25 μm) of each micropost and X is the tip displacement of the micropost (Fig. 5A). Since the microposts are fabricated in rectangular instead of cylindrical, the rectangle section modulus is:
(5)
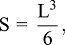
which substitutes the section modulus of the circular section:
(6)
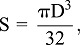
to generate the equation for calculating the twitch force of bending rectangular post:
(7)
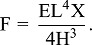
After the values of the parameter E, L and H are applied in Eqn 7, the formula for calculating twitch force of bending rectangular post becomes:
(8)
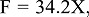
where F and X are in the scales of nano-newton (nN) and micrometer (μm), respectively.
